# Randomised clinical trial: the effects of a multispecies probiotic vs. placebo on innate immune function, bacterial translocation and gut permeability in patients with cirrhosis

**DOI:** 10.1111/apt.13788

**Published:** 2016-09-04

**Authors:** A. Horvath, B. Leber, B. Schmerboeck, M. Tawdrous, G. Zettel, A. Hartl, T. Madl, S. Stryeck, D. Fuchs, S. Lemesch, P. Douschan, E. Krones, W. Spindelboeck, F. Durchschein, F. Rainer, G. Zollner, R. E. Stauber, P. Fickert, P. Stiegler, V. Stadlbauer

**Affiliations:** ^1^ Department of Gastroenterology and HepatologyMedical University of GrazGrazAustria; ^2^ Department of Transplantation SurgeryMedical University of GrazGrazAustria; ^3^ Centre for Biomarker Research in Medicine (CBmed)GrazAustria; ^4^ Institute of Molecular Biology and BiochemistryMedical University of GrazGrazAustria; ^5^ Division of Biological ChemistryBiocentreMedical University of InnsbruckInnsbruckAustria

## Abstract

**Background:**

Probiotics may correct intestinal dysbiosis and proinflammatory conditions in patients with liver cirrhosis.

**Aim:**

To test the effects of a multispecies probiotic on innate immune function, bacterial translocation and gut permeability.

**Methods:**

In a randomised, double blind, placebo‐controlled study, stable cirrhotic out‐patients either received a daily dose of a probiotic powder containing eight different bacterial strains (Ecologic Barrier, Winclove, Amsterdam, The Netherlands) (*n* = 44) or a placebo (*n* = 36) for 6 months and were followed up for another 6 months.

**Results:**

We found a significant but subclinical increase in neutrophil resting burst (2.6–3.2%, *P* = 0.0134) and neopterin levels (7.7–8.4 nmol/L, *P* = 0.001) with probiotics but not with placebo. Probiotic supplementation did not have a significant influence on neutrophil phagocytosis, endotoxin load, gut permeability or inflammatory markers. Ten severe infections occurred in total; one during intervention in the placebo group, and five and four after the intervention has ended in the probiotic and placebo group, respectively. Liver function showed some improvement with probiotics but not with placebo.

**Conclusions:**

Probiotic supplementation significantly increased serum neopterin levels and the production of reactive oxygen species by neutrophils. These findings might explain the beneficial effects of probiotics on immune function. Furthermore, probiotic supplementation may be a well‐tolerated method to maintain or even improve liver function in stable cirrhosis. However, its influence on gut barrier function and bacterial translocation in cirrhotic patients is minimal.

## Introduction

Liver cirrhosis is the 9th biggest burden of disease in Europe and the 11th most common cause of death in the US.^1^, ^2^ Bacterial infections are common complications of cirrhosis resulting from significantly impaired immune responses. Among others, oxidative burst, phagocytosis and migration of neutrophils are dysfunctional in these patients^3^, ^4^, ^5^, ^6^, ^7^ and have been linked to elevated infection rates and poor survival.^8^, ^9^

A proposed hypothesis for the origin of neutrophil dysfunction in cirrhosis assumes a disrupted intestinal barrier through which luminal, microflora‐derived endotoxin can enter the portal blood stream. Limited filter capacities of the cirrhotic liver and formation of portosystemic shunts cause an endotoxin overflow into the systemic blood.^10^ Endotoxin acts as a potent priming agent for neutrophils and leads to production of small amounts of reactive oxygen species as an anti‐microbial strategy.^11^ Additionally, the presence of endotoxin in the blood stream increases bacterial clearance.^12^ However, chronic over‐stimulation might lead to exhaustion, leaving the neutrophils disabled to react to pathogens. Consequently, phagocytic capacity and oxidative burst would be reduced and infection rates increased.

Probiotic supplementation is already recommended for several gut‐related diseases and allergies.^13^ Clinical trials show probiotic preparations to improve liver function in different stages of alcoholic liver disease.^14^, ^15^, ^16^, ^17^ Additionally, potential beneficial effects of probiotics on neutrophil phagocytosis have been attested for alcoholic cirrhosis in a short‐term, open‐label study.^18^ Therefore, we hypothesised that the administration of a multispecies probiotic over 6 months decreases intestinal permeability, reduces bacterial translocation and consequently restores and maintains neutrophil function in stable cirrhosis of different aetiologies. To test this hypothesis, we designed and conducted this randomised, double blind, placebo‐controlled trial testing the effects of Ecologic Barrier (Winclove, Amsterdam, The Netherlands) in patients with liver cirrhosis.

## Methods

### Patients and study design

Between July 2012 and September 2013, patients from the out‐patient clinic at the Department of Gastroenterology and Hepatology or the Department of Transplantation Surgery, both University Hospital Graz, with cirrhosis of any aetiology between the age of 18 and 80 years were eligible for the study if none of the following conditions applied: Child–Pugh score 12 or higher, alcohol abuse within 2 weeks prior to inclusion, active infection at screening, antibiotic therapy except for permanent prophylaxis, simultaneous intake of pro‐/pre‐/symbiotic, gastrointestinal haemorrhage within 2 weeks prior to inclusion, immunomodulation drugs, hepatic encephalopathy stage two or higher, renal failure (creatinine over 1.7 mg/dL), pancreatitis, other severe diseases unrelated to cirrhosis, malignancy, suspected noncompliance, pregnancy. Diagnosis of cirrhosis was based on liver histology or characteristic clinical and radiological features. After giving written informed consent, eligible patients were randomised in a 1:1 ratio to one of two parallel groups (permutated blocks, Randomizer software, Institute of Medical Informatics, Medical University of Graz) and stratified for aetiology and permanent antibiotic use. Patients were included in the study for 12 months. In the first 6 months, they received either a daily dose of a multispecies probiotic (6 g, 2.5 × 10^9^ CFU/g) or a placebo. Patients were clinically observed for another 6 months. Detailed assessments were performed after 3 months of intervention, after 6 months of intervention (end of intervention) and after further 6 months of observation (end of observation). The *a priori* primary end point was the change in phagocytic capacity of neutrophils between baseline and 6 months, and secondary endpoints were numbers of clinically relevant infections, neutrophil oxidative burst, endotoxin levels, inflammatory responses and gut permeability. Infections were considered as mild when patients did not need hospitalisation, whereas severe infections required hospitalisation.

The probiotic consisted of *Bifidobacterium bifidum* W23, *Bifidobacterium lactis* W52, *Lactobacillus acidophilus* W37, *Lactobacillus brevis* W63, *Lactobacillus casei* W56, *Lactobacillus salivarius* W24, *Lactococcus lactis* W19 and *Lactococcus lactis* W58 and is marketed as Ecologic Barrier in The Netherlands and as Omnibiotic Hetox in Austria, Germany and Switzerland. The product was chosen based on *in vitro* properties and relatively high amount of bacteria.^19^ Patients, caregivers, investigators and outcome assessors were blinded to the allocation. The study medication was concealed in blank sachets identified by consecutive numbers. An allocation list was kept by an independent trial pharmacist and disclosed after the last patient has finished the study. Adherence to study medication was measured by counting empty and remaining sachets the patients returned to the study nurse. During regular phone calls, patients were asked whether they take the study medication and were encouraged to adhere to the protocol. Sample size calculation was based on improvement of phagocytic capacity (pre‐specified primary outcome) as assessed in a preceding study (percentage of healthy control values of geometric mean fluorescence intensity of fluorescein isothiocyanate‐positive cells).^18^ According to these data, an improvement of 25 percentage points in phagocytic capacity was anticipated. With an alpha of 0.05 and a beta of 0.2, assuming a 20% dropout rate, 92 patients were needed for the study (46 in each arm). In addition, healthy controls (*n* = 51) were included in the study. The study protocol was approved by the regional ethics committee in Graz (23‐096 ex 10/11), registered at clinicaltrials.gov (NCT01607528), and performed according to the Declaration of Helsinki.

Phagocytosis and burst function was assessed by Phagotest/Bursttest (Glycotope, Heidelberg, Germany). Neopterin, a marker for macrophage activation and modulator of neutrophil function,^20^ was assessed by enzyme‐linked immunosorbent assay. Gut permeability was assessed by a panel of markers: Zonulin and calprotectin in stool, diamine oxidase in serum, sucrose recovery and lactulose–mannitol ratio. Differential sugar absorption test was analysed by nuclear magnetic resonance spectroscopy. Serum and stool markers were measured by enzyme‐linked immunosorbent assay, and endotoxin was determined with adapted HEK‐Blue LPS Detection Kit (Invivogen, San Diego, CA, USA). For further information, see Supporting information.

### Statistical analysis

Statistical per protocol analysis was performed with spss 21 (IBM Germany GmbH, Ehningen, Germany). Between‐group differences of categorical variables were assessed by chi‐squared test/Fisher's exact test. Between‐group differences of continuous variables were assessed by Mann–Whitney/Wilcoxon signed rank tests and Kruskal–Wallis/Friedman tests for unpaired/paired data to compare two or more groups respectively. Multiple comparisons with Bonferroni correction were used as *post hoc* tests. All tests were performed on a 5% significance level. As intention‐to‐treat analysis gave the same significant changes (neopterin and neutrophil oxidative burst) within the groups as per protocol analysis, we decided to show per protocol analysis in the presented study (see also Supporting information).

## Results

### Patients' and baseline characteristics

**Figure 1:**
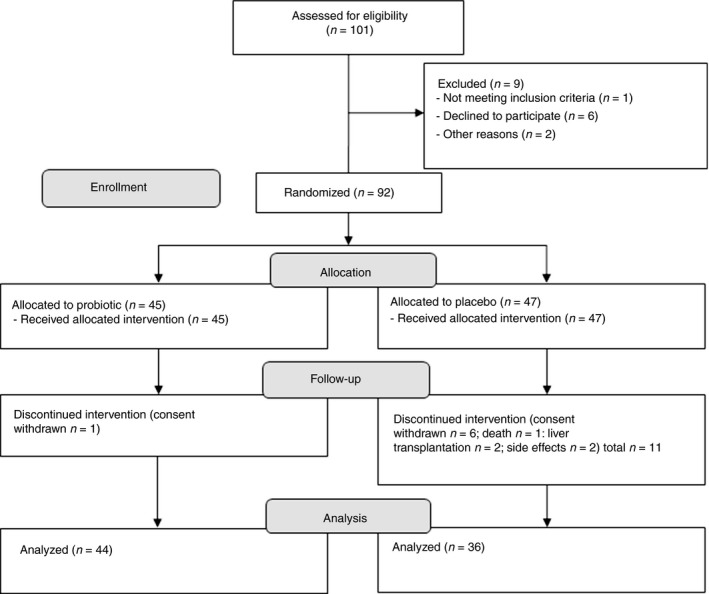
CONSORT flow diagram.

Between July 2012 and September 2013 in total 101 patients were screened for eligibility, 92 were included and 80 finished the study (Figure 1). For baseline characteristics, see Tables 1 and S1. Patients in the two study groups were comparable regarding age, sex, aetiology and severity of liver disease. However, after exclusion of dropouts, liver function was significantly worse in the probiotic compared to the placebo group (Child–Pugh score *P* = 0.02 and MELD score *P* = 0.05) at baseline. Two patients received antibiotic prophylaxis; one was randomised into the probiotic group and one into the placebo group. Albumin levels remained constant over the intervention period and decreased slightly at the end of observation (*P* = 0.007). There were no changes in the placebo group. Mild ascites, hepatic encephalopathy, alanine aminotransferase (ALT), aspartate aminotransferase (AST), creatinine, bilirubin, gamma glutamyl transferase, triglycerides, prothrombin time international normalised ratio, neutrophil and monocyte count remained unchanged in both groups (Tables 1 and S1).

**Table 1:** Patients' characteristics and routine laboratory measurements for liver cirrhosis before, during and after intervention with a multispecies probiotic for the probiotic and placebo group compared to healthy controls

	Group	Patients: probiotics (*n* = 44) and placebo (*n* = 36)				Controls (*n* = 51)
		Baseline	3 months of intervention	End of intervention (6 months)	End of observation (12 months)	
Age (years)	Probiotics	60 (54; 64)*	–	–	–	53 (44; 60)
	Placebo	56 (50; 63)	–	–	–	
Sex (M/F)	Probiotics	32/12*	–	–	–	22/29
	Placebo	26/10*	–	–	–	
Aetiology (Alc/HCV/others)	Probiotics	24/8/12	–	–	–	–
	Placebo	20/5/11	–	–	–	
CPG (A/B+C)	Probiotics	28/16†	31/13† ^,^ §	29/15† ^,^ §	24/20†	–
	Placebo	33/3† ^,^ §	33/3† ^,^ §	33/3† ^,^ §	33/3† ^,^ §	
CPS	Probiotics	6 (5; 7)†	5 (5; 7)†	6 (5; 7)†	7 (5; 7)†	–
	Placebo	5 (5; 6)	5 (5; 6)	5 (5; 6)	5 (5; 6)	
MELD	Probiotics	12 (9; 15)†	11 (9; 14)†	11 (9; 15)†	12 (9; 14)†	–
	Placebo	9 (8; 13)	9 (8; 11)	9 (8; 12)	8 (7; 11)	
ALT (U/L)	Probiotics	36.5 (27.0; 51.25)*	34.5 (27.5; 48.5)	38.5 (25.8; 52.3)	36.0 (26.0; 53.5)	21.0 (16.5; 27.5)
	Placebo	32.5 (20.75; 46.25)*	30.0 (22.0; 43.3)	29.5 (22.0; 49.8)	30.0 (22.0; 42.5)	
AST (U/L)	Probiotics	49.0 (37.75; 69.5)*	44.5 (36.0; 59.0)	53.5 (36.8; 70.0)†	49.5 (37.5; 68.3)	22.0 (19.0; 27.0)
	Placebo	42.5 (32.5; 56.5)*	40.5 (31.5; 58.0)	37.5 (30.8; 59.0)	45.0 (29.8; 63.3)	
Crea (mg/dL)	Probiotics	0.85 (0.73; 0.96)	0.83 (0.73; 0.94)	0.85 (0.74; 1.01)	0.85 (0.75; 0.98)	0.86 (0.77; 1.0)
	Placebo	0.81 (0.72; 0.94)	0.78 (0.70; 0.98)	0.80 (0.70; 0.88)	0.83 (0.71; 0.92)	
Alb (g/dL)	Probiotics	4.0 (3.3; 4.5)* ^,^ ‡	4.0 (3.4; 4.5)†	4.0 (3.4; 4.5)†	3.9 (3.3; 4.4)†	4.5 (4.4; 4.8)
	Placebo	4.3 (4.1; 4.7)	4.4 (4.0; 4.6)	4.3 (4.0; 4.4)	4.3 (3.9; 4.5)	
Bili (mg/dL)	Probiotics	1.38 (0.78; 2.41)*	1.29 (0.74; 2.25)	1.32 (0.77; 2.69)	1.46 (0.88; 2.41)†	0.50 (0.38; 0.61)
	Placebo	1.11 (0.63; 1.42)*	0.97 (0.74; 1.38)	0.95 (0.68; 1.48)	1.00 (0.64; 1.59)	
INR	Probiotics	1.27 (1.14; 1.43)*	1.27 (1.18; 1.39)†	1.28 (1.16; 1.48)†	1.30 (1.14; 1.45)†	1.01 (0.98; 1.05)
	Placebo	1.20 (1.12; 1.27)*	1.18 (1.09; 1.32)	1.18 (1.11; 1.25)	1.14 (1.09; 1.25)	

Alc, alcoholic cirrhosis; HCV, hepatitis C virus‐associated cirrhosis; CPG, Child–Pugh grade; CPS, Child–Pugh score; MELD, model of end‐stage liver disease; ALT, alanine aminotransferase, AST, aspartate transaminase; Crea, creatinine; Alb, albumin; Bili, total bilirubin; INR, prothrombin time international normalised ratio.

Data are given in median (Q1; Q3).

*Significant difference compared to control group; †significant difference between test groups; ‡significant change over time; §to expected distribution; significance level 0.05.

### Excellent compliance and unremarkable adverse event profile

Compliance to the study was excellent. There were significantly less dropouts in the probiotic group than expected and considered in the sample size calculation (1 vs. 9, *P* = 0.007) and significantly less compared to the placebo group (1 and 11, *P* = 0.003). Dropout rate in the placebo group was within the expected range. Ten out of 12 dropouts left the study before the end of treatment. Reasons for dropout were in the probiotic group withdrawal of consent (*n* = 1), in the placebo group withdrawal of consent (*n* = 6), accident‐related death (*n* = 1), liver transplantation (*n* = 2) and suspected adverse events (*n* = 2, epistaxis with pre‐existing arterial hypertension and nausea/flatulence). Adherence to the study medication was remarkable (176 and 179 of 180 scheduled doses of probiotic and placebo respectively). Adverse events possibly related to the study product were flatulence, gastric pain, diarrhoea and nausea, and affected 41% of patients in the probiotic group and 33% in the placebo group (*P* = 0.48) during the initial phase of intervention. Dietary habits did not change throughout the study in both groups.

### Neutrophil phagocytosis decreased over time irrespective of intervention

**Figure 2:**
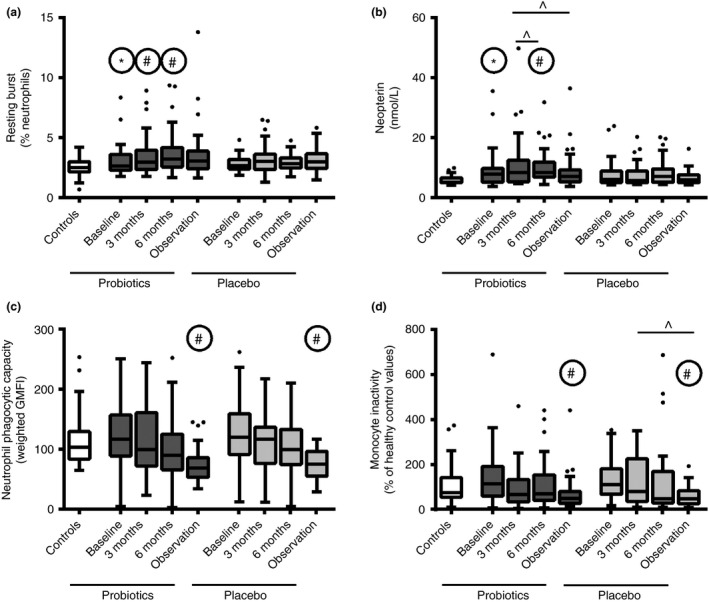
Effects of a multispecies probiotic on innate immune function before, during and after intervention for test groups compared to healthy controls. (a) Oxidative resting burst of neutrophils; (b) serum levels of neopterin; (c) Phagocytic capacity of neutrophils; (d) Inactive monocytes. *Significant differences to healthy controls; #significant differences to according baseline. ^Significant changes between indicated groups; significance level 0.05.

Baseline neutrophil phagocytosis in both groups was comparable to controls. Probiotics had no effect on neutrophil phagocytosis (primary endpoint). In both groups, phagocytic capacity decreased significantly over time (*P* < 0.001), while the population of inactive neutrophils stayed on a high but constant level. The phagocytic capacity of monocytes was not impaired at baseline but a slight increase could be found after 3 months of probiotics; placebo showed no effect. Monocyte inactivity decreased significantly in both groups during the study, possibly to balance neutrophil dysfunction (Figures 2 and S1).

### Anti‐microbial activity improved during probiotic intervention

During intervention, neutrophil resting burst increased significantly in the probiotic group (2.6–3.2% after 6 months, *P* = 0.0134) but not in the placebo group (Figure 2). Otherwise oxidative burst profile was comparable between groups (Figure S1). Neopterin has been shown to induce the production of reactive species in neutrophils.^20^ Serum neopterin levels were significantly increased in the probiotic group compared to controls at baseline. It further increased at the end of intervention in the probiotic group (*P* = 0.004) and decreased to baseline values at the end of observation (*P* = 0.004). No significant changes were found in the placebo group (Figure 2). Other anti‐microbial molecules and acute phase proteins were unaffected (Table S2).

As both reactive oxygen species and neopterin have anti‐microbial properties, we tested the bactericidal ability of serum, which was significantly impaired in both patient groups compared to controls. No improvement could be detected during probiotic intervention or placebo in the whole study cohort (Table S2). However, in the subgroup of alcoholic cirrhosis, 6 months of probiotics tended to improve serum killing capacity compared to baseline. There was no change in the placebo group (Figure S1).

### Low infection rates in both study groups

Infection rates were exceptionally low in both groups. We observed a numerical trend towards lower frequency of mild infections in the probiotic group compared to the placebo group (15 vs. 28 during intervention, 6 vs. 11 in the observation period). Severe infections, defined by hospitalisation of the patient, were rare. During intervention, no severe infection occurred in the probiotic group but one in the placebo group. During observation, severe infections increased to five in the probiotic and four in the placebo group. The occurrence of severe infection coincided with the deterioration of phagocytic capacity of neutrophils.

### Impaired gut barrier integrity was not restored by probiotic treatment

Gut barrier integrity was significantly impaired in the test groups compared to healthy controls at baseline (lactulose–mannitol ratio, diamine oxidase and calprotectin). Probiotic intervention showed no significant effect on those markers. In the probiotic group, mannitol recovery was stable throughout the intervention, while in the placebo group, mannitol recovery decreased significantly during intervention and further during the observation period. However, these changes did not impact on lactulose–mannitol ratio. Zonulin and sucrose recovery did not differ between groups, compared to controls or over time (Figure S2 and Table S3).

### Probiotics did not change endotoxin load, endotoxin‐related proteins and serum cytokine levels

Endotoxin load was significantly higher in patients compared to controls (*P* < 0.001). In both groups, endotoxin levels decreased slightly over time (*P* = 0.29 and *P* = 0.05, probiotic and placebo respectively) (Figure S2). A correlation between endotoxin levels and neutrophils phagocytic capacity could not be found (*r* = 0.06; *P* = 0.57). Endotoxin‐related proteins, soluble cluster of differentiation 14 and LPS‐binding protein were slightly elevated in patients compared to controls. No changes over time could be observed (Table S3). Interleukin‐8 and ‐10, but not tumour necrosis factor α, interleukin‐1b or ‐6, were significantly increased in patients compared to controls. Probiotics had no effect on any cytokine tested (data not shown).

### Improvement of liver function during probiotic intervention

Of the 16 patients in the probiotic group with a Child–Pugh score of 7 or higher, six improved after initiation of probiotics, seven did not change and three deteriorated. In the placebo group, only three patients had a Child–Pugh score of 7 or higher; one stayed unchanged, one improved, one deteriorated (four patients dropped out) (Figure S3). These results are reflected in a slight improvement in MELD score in the probiotic group (*P* = 0.09). No change in liver function was found in the placebo group (Table 1). However, due to the higher dropout rate in the placebo group, a representative comparison was not possible.

## Discussion

We tested a multispecies probiotic in a cohort of stable cirrhotics to investigate its effect on innate immunity and gut permeability. Although the primary endpoint could not be achieved, we found that administration of the probiotic for 6 months significantly improved anti‐microbial activity and slightly improved liver function.

The importance of humoral anti‐microbial responses has long been recognised and gained more significance in the light of emerging antibiotic resistance problems.^21^, ^22^, ^23^, ^24^ Neopterin is a marker for macrophage activation. It is produced by macrophages upon stimulation and has anti‐microbial and antioxidant properties.^25^ The increase in neopterin levels in our study cohort was minimal and well below the levels reported for malignancies.^26^ Nevertheless, given the regulatory properties of this molecule, a subclinical elevation of macrophage activity during probiotic intervention might indicate a potentially beneficial activation of the immune system, e.g., neopterin was found to amplify the generation of anti‐microbial reactive species in cultured granulocytes.^20^ This was reflected in a significant increase in neutrophil resting burst in our study. This increase induced by probiotics was small and well below the level of resting burst seen in decompensated cirrhosis or alcoholic hepatitis.^8^ Active infection was excluded as cause for the activation because of normal levels of acute phase proteins as well as temperature, heart rate and respiratory rate (data not shown) during the study. A moderate activation of immune cells in resting conditions might give a clearance benefit in early stages of active infections. Activated or primed phagocytes have been shown to clear pathogens more effectively from the blood stream than resting cells.^12^ We found that especially the subgroup of alcoholic cirrhotics might benefit from probiotic supplementation in regard to improved serum killing incapacity. Furthermore, cytokine levels, acute phase proteins, liver transaminases and the beneficial effect on liver function do not suggest harmful effects. However, more specialised studies are needed to acquire conclusive evidence and to elucidate the full physiological spectrum of neopterin in liver disease. To date, the clinical significance of the improvement in neopterin and neutrophil resting burst is not clear and therefore has to be evaluated in further studies.

Cirrhotic patients suffer from a high risk of bacterial infection (4–54%) including spontaneous bacterial peritonitis, pneumonia, urinary tract infections and bacteraemia.^27^, ^28^, ^29^, ^30^ Probiotics were shown to decrease infection rates in liver transplant recipients in a placebo‐controlled, double blind study.^31^ In our study, infection rates during intervention were exceptionally low in both the probiotic and placebo group. Only one patient in the placebo group was hospitalised because of a bacterial infection. In the observation period, severe infections occurred more often (7% of all patients); however, infection rates were still low compared to the literature.^27^ This might be due to relatively stable cirrhosis states as patients were recruited from our out‐patient clinic. Regarding mild infections, we found a tendency to a reduced occurrence in the probiotic group. However, the documentation of mild infections relayed on self‐reporting and was therefore prone to bias.

A substantial amount of bacterial infections in cirrhosis result from the translocation of intestinal bacteria. Bacterial translocation through a damaged intestinal barrier was reported for decompensated cirrhotic patients.^32^ In our study, we also confirmed increased gut permeability in stable cirrhosis. The alterations point to an increased permeability and inflammation in the small intestine but not in the gastroduodenal part of the gastrointestinal tract. The beneficial effect of probiotics on gut permeability has been shown *in vitro*,* in vivo* and in humans.^19^, ^33^, ^34^ We found that probiotics maintained mannitol recovery, while it gradually decreased in the placebo group. Reduced mannitol absorption might be due to reduced intestinal surface in cirrhosis.^35^ Therefore, probiotics might have a beneficial effect on the intestinal epithelium. Elevated levels of zonulin in stool were associated with increased gut permeability.^36^ Although patients in our study showed an increase in various other permeability markers, zonulin remained within normal range. Low levels of zonulin in patients with high gut permeability might stem from the loss of epithelial cells common in cirrhosis.

Probiotic administration was shown to decrease endotoxin levels in several studies.^37^ In our study, no treatment‐related changes in endotoxin load occurred. No association between endotoxin levels and liver function, gut permeability or endotoxin‐related proteins could be found. Upregulation of endotoxin‐related proteins was not observed in our study probably due to relatively low levels of endotoxin and stable liver function.

No beneficial effect on neutrophil phagocytosis could be observed. To confirm the decrease of neutrophil phagocytosis over time, we conducted cross‐incubation experiments (data not shown) and similar changes in phagocytic function could be observed when neutrophils of healthy volunteers were incubated with serum of patients, especially in more advanced cirrhosis. Serum‐facilitated neutrophil dysfunction has already been shown for alcoholic hepatitis.^7^ Elevated endotoxin levels were proposed to be the cause of impaired phagocytosis of neutrophils. However, we found no association between those parameters. According to this study, the association between neutrophil dysfunction and endotoxin is not a driving force in immune deficiency of stable cirrhotics. We found a strong association between aetiology and neutrophil function but not between aetiology and endotoxin levels. Cytokine levels were unaffected by probiotic administration and changes in cytokine levels that has been reported in other publications could not be reproduced.^18^, ^38^

In our study, patients in the probiotic group with Child–Pugh grades 7 or higher were more likely to improve than to deteriorate in their Child–Pugh score. The beneficial effects on liver function were in accordance to an increasing body of literature.^14^, ^15^, ^16^, ^17^ As therapeutic interventions to improve liver function in cirrhosis are an unmet clinical need, probiotics are an attractive and safe option to consider.

Compliance in our study was excellent in both groups which was consistent with low rates of side effects. Side effects were mild and most symptoms were gastrointestinal complaints, as expected with probiotics. Most of the side effects appeared in the first 2 weeks of the intervention and subsided after that. High adherence to the study protocol and significantly lower dropout rate in the probiotic group suggest an improvement in the overall well‐being of patients by the probiotics.

Some safety concerns regarding the use of probiotics in patients with increased gut permeability and/or compromised immune system had been raised.^39^ However, to date, no reports about severe side effects of probiotics in cirrhosis have emerged.^40^ Data from our trial suggest that a multispecies probiotic is safe to use in cirrhotic patients. We did not observe any severe adverse events, no severe infections and no increase in acute phase proteins during the intervention with the probiotic. Also, the slightly reduced rates of mild infections in the probiotic group did not give any indication for safety concerns.

Our study has some limitations: The study was performed as a single centre study; however, we managed to recruit a relatively large number of patients with different aetiologies. Due to the higher dropout rate in the placebo group and the fact that mainly patients with higher Child–Pugh score terminated the study early, the liver function in the probiotic group was worse compared to the placebo group in the per protocol analysis. The effect of the probiotic supplement on the gut microflora has not been evaluated yet. Therefore, it is unclear at present whether the intervention changed the microbiome *per se*. Although the probiotic strains used in this study were carefully selected based on their *in vitro* characteristics, the primary endpoint of this study could not be achieved. It is however possible that an improved product would result in a different outcome. Measurement of gut permeability in humans is always a matter of debate^41^; therefore, we chose five different parameters. The measurement of endotoxin in biological fluids also poses a methodological problem. The standard method (Limulus amoebocyte lysate assay) is not designed to detect endotoxin in serum samples. In addition, several other issues have been raised with this test.^42^ Bound endotoxin remains undetected, even if biologically active. The cell‐based method used in this study mimics bio‐availability of endotoxin more accurately than the limulus amoebocyte lysate assay.^43^

In conclusion, probiotics were very well tolerated by the patients and side effects were rare. Probiotic supplementation significantly increased serum neopterin levels and the production of reactive oxygen species by neutrophils. These findings might explain the beneficial effects of probiotics on immune function. Markers of gut permeability and translocation of Gram‐negative bacteria assessed in this study showed no improvement and neutrophil phagocytosis worsened irrespective of the intervention over the course of 1 year to only 60% of the baseline value. Liver function was slightly improved during intervention and decreased without it. Further randomised, controlled studies – ideally in a multicentre setting – are necessary to confirm the beneficial effect on anti‐microbial activity and liver function.

## Authorship

*Guarantor of the article*: Angela Horvath, Vanessa Stadlbauer.

*Author contributions*: VS and BL designed the study, VS, PD, EK, WS, FD, FR, GZ, GZ, RES, PF and PS recruited the patients, performed the clinical assessments and collected outcome data, AH, BL, BS, MT, AH, TM, SS, DF and SL performed the laboratory assays. AH analysed the data, VS and AH interpreted the data and wrote the first draft of the manuscript. AH and VS had access to all of the data and can vouch for the integrity of the data analyses. All authors revised the manuscript critically for intellectual content, and have approved the final version.

## Supporting information


**Data S1.** Rationale for per protocol analysis and additional methods.
**Table S1.** Patients' characteristics and routine laboratory measurements for liver cirrhosis before, during and after intervention with a multispecies probiotic for the probiotic and placebo group compared to healthy controls.
**Table S2.** Anti‐microbial molecules, acute‐phase proteins and serum killing incapacity before, during and after probiotic intervention for the probiotic and placebo group compared to healthy controls.
**Table S3.** Parameters of endotoxin binding and gut permeability before, during and after probiotic intervention for the probiotic and placebo group compared to healthy controls.
**Figure S1.** Effects of a multispecies probiotic on innate immune function before, during and after intervention for test groups compared to healthy controls. A+B. Oxidative burst profile of neutrophils during probiotic or placebo administration; C. Phagocytic capacity of monocytes; D. Inactive neutrophils; E. Serum killing incapacity of alcoholic cirrhotics; F. Change in serum killing incapacity of alcoholic cirrhotics after 6 months of intervention. *Significant differences to healthy controls; #significant differences to according baseline; significance level 0.05.
**Figure S2.** Gut permeability and bacterial translocation before, during and after intervention for test groups compared to healthy controls. A, B. Mannitol and lactulose recovery in urine after triple sugar ingestion. C. Lactulose–mannitol ratio. D. Calprotectin in stool. E. Diamine oxidase in serum. F. Endotoxin in serum. *Significant differences to healthy controls; #significant differences to according baseline; ^significant changes between indicated groups; significance level 0.05.
**Figure S3.** A. Formation of low and high phagocytic neutrophil population as used to calculate phagocytic capacity. B. Changes in Child–Pugh score from baseline to end of treatment in probiotic and placebo group; thickness of bars corresponds to number of patients with respective changes.

## References

[1] Murray CJ , Lopez AD . Measuring the global burden of disease. N Engl J Med 2013; 369: 448–57. 23902484 10.1056/NEJMra1201534

[2] WHO . The global burden of disease. 2004 Update, 2008.

[3] Shawcross DL , Wright GA , Stadlbauer V , *et al* Ammonia impairs neutrophil phagocytic function in liver disease. Hepatology 2008; 48: 1202–12. 18697192 10.1002/hep.22474

[4] Taylor NJ , Nishtala A , Manakkat Vijay GK , *et al* Circulating neutrophil dysfunction in acute liver failure. Hepatology 2013; 57: 1142–52. 23079896 10.1002/hep.26102

[5] Tritto G , Bechlis Z , Stadlbauer V , *et al* Evidence of neutrophil functional defect despite inflammation in stable cirrhosis. J Hepatol 2011; 55: 574–81. 21236309 10.1016/j.jhep.2010.11.034

[6] Nieto JC , Sanchez E , Romero C , *et al* Impaired innate immune response of leukocytes from ascitic fluid of patients with spontaneous bacterial peritonitis. J Leukocyte Biol 2015; 98: 819–25. 26254307 10.1189/jlb.3AB0315-106R

[7] Stadlbauer V , Mookerjee RP , Wright GA , *et al* Role of toll‐like receptors 2, 4, and 9 in mediating neutrophil dysfunction in alcoholic hepatitis. Am J Physiol Gastrointest Liver Physiol 2009; 296: G15–22. 19033535 10.1152/ajpgi.90512.2008 PMC2636930

[8] Mookerjee RP , Stadlbauer V , Lidder S , *et al* Neutrophil dysfunction in alcoholic hepatitis superimposed on cirrhosis is reversible and predicts the outcome. Hepatology 2007; 46: 831–40. 17680644 10.1002/hep.21737

[9] Taylor NJ , Manakkat Vijay GK , Abeles RD , *et al* The severity of circulating neutrophil dysfunction in patients with cirrhosis is associated with 90‐day and 1‐year mortality. Aliment Pharmacol Ther 2014; 40: 705–15. 25060167 10.1111/apt.12886

[10] Wiest R , Garcia‐Tsao G . Bacterial translocation (BT) in cirrhosis. Hepatology 2005; 41: 422–33. 15723320 10.1002/hep.20632

[11] Lamb FS , Hook JS , Hilkin BM , Huber JN , Volk AP , Moreland JG . Endotoxin priming of neutrophils requires endocytosis and NADPH oxidase‐dependent endosomal reactive oxygen species. J Biol Chem 2012; 287: 12395–404. 22235113 10.1074/jbc.M111.306530 PMC3320989

[12] Balmer ML , Slack E , deGottardi A , *et al* The liver may act as a firewall mediating mutualism between the host and its gut commensal microbiota. Science Transl Med 2014; 6: 237ra66. 10.1126/scitranslmed.3008618 24848256

[13] Floch MH , Walker WA , Madsen K , *et al* Recommendations for probiotic use‐2011 update. J Clin Gastroenterol 2011; 45(Suppl.): S168–71. 21992958 10.1097/MCG.0b013e318230928b

[14] Liu Q , Duan ZP , Ha DK , Bengmark S , Kurtovic J , Riordan SM . Synbiotic modulation of gut flora: effect on minimal hepatic encephalopathy in patients with cirrhosis. Hepatology 2004; 39: 1441–9. 15122774 10.1002/hep.20194

[15] Lata J , Novotny I , Pribramska V , *et al* The effect of probiotics on gut flora, level of endotoxin and Child‐Pugh score in cirrhotic patients: results of a double‐blind randomized study. Eur J Gastroenterol Hepatol 2007; 19: 1111–3. 17998837 10.1097/MEG.0b013e3282efa40e

[16] Dhiman RK , Rana B , Agrawal S , *et al* Probiotic VSL#3 reduces liver disease severity and hospitalization in patients with cirrhosis: a randomized, controlled trial. Gastroenterology 2014; 147: 1327–37.e3. 25450083 10.1053/j.gastro.2014.08.031

[17] Kirpich IA , Solovieva NV , Leikhter SN , *et al* Probiotics restore bowel flora and improve liver enzymes in human alcohol‐induced liver injury: a pilot study. Alcohol 2008; 42: 675–82. 19038698 10.1016/j.alcohol.2008.08.006 PMC2630703

[18] Stadlbauer V , Mookerjee RP , Hodges S , Wright GA , Davies NA , Jalan R . Effect of probiotic treatment on deranged neutrophil function and cytokine responses in patients with compensated alcoholic cirrhosis. J Hepatol 2008; 48: 945–51. 18433921 10.1016/j.jhep.2008.02.015

[19] Van Hemert S , Ormel G . Influence of the multispecies probiotic Ecologic^®^ BARRIER on parameters of intestinal barrier function. Food Nutr Sci 2014; 5: 1739–45.

[20] Razumovitch JA , Fuchs D , Semenkova GN , Cherenkevich SN . Influence of neopterin on generation of reactive species by myeloperoxidase in human neutrophils. Biochim Biophys Acta 2004; 1672: 46–50. 15056492 10.1016/j.bbagen.2004.02.007

[21] Zangenah S , Bergman P . Rapid killing of *Capnocytophaga canimorsus* and *Capnocytophaga cynodegmi* by human whole blood and serum is mediated via the complement system. SpringerPlus 2015; 4: 517. 26405637 10.1186/s40064-015-1308-9 PMC4574033

[22] Homann C , Varming K , Hogasen K , *et al* Acquired C3 deficiency in patients with alcoholic cirrhosis predisposes to infection and increased mortality. Gut 1997; 40: 544–9. 9176087 10.1136/gut.40.4.544 PMC1027133

[23] Luthje P , Brauner A . Novel strategies in the prevention and treatment of urinary tract infections. Pathogens 2016; 5: doi: 10.3390/pathogens5010013. 10.3390/pathogens5010013 PMC4810134 26828523

[24] Lamontagne A , Long RE , Comunale MA , *et al* Altered functionality of anti‐bacterial antibodies in patients with chronic hepatitis C virus infection. PLoS ONE 2013; 8: e64992. 23750224 10.1371/journal.pone.0064992 PMC3672197

[25] Hoffmann G , Wirleitner B , Fuchs D . Potential role of immune system activation‐associated production of neopterin derivatives in humans. Inflamm Res 2003; 52: 313–21. 14504669 10.1007/s00011-003-1181-9

[26] Hamerlinck FFV . Neopterin: a review. Exp Dermatol 1999; 8: 167–76. 10389633 10.1111/j.1600-0625.1999.tb00367.x

[27] Leber B , Spindelboeck W , Stadlbauer V . Infectious complications of acute and chronic liver disease. Semin Respir Crit Care Med 2012; 33: 80–95. 22447263 10.1055/s-0032-1301737

[28] Tandon P , Garcia‐Tsao G . Bacterial infections, sepsis, and multiorgan failure in cirrhosis. Semin Liver Dis 2008; 28: 26–42. 18293275 10.1055/s-2008-1040319

[29] Guerrero Hernandez I , Torre Delgadillo A , Vargas Vorackova F , Uribe M . Intestinal flora, probiotics, and cirrhosis. Ann Hepatol 2008; 7: 120–4. 18626428

[30] Jalan R , Fernandez J , Wiest R , *et al* Bacterial infections in cirrhosis: a position statement based on the EASL Special Conference 2013. J Hepatol 2014; 60: 1310–24. 24530646 10.1016/j.jhep.2014.01.024

[31] Rayes N , Seehofer D , Theruvath T , *et al* Supply of pre‐ and probiotics reduces bacterial infection rates after liver transplantation–a randomized, double‐blind trial. Am J Transplant 2005; 5: 125–30. 15636620 10.1111/j.1600-6143.2004.00649.x

[32] Cirera I , Bauer TM , Navasa M , *et al* Bacterial translocation of enteric organisms in patients with cirrhosis. J Hepatol 2001; 34: 32–7. 11211904 10.1016/s0168-8278(00)00013-1

[33] Chen P , Torralba M , Tan J , *et al* Supplementation of saturated long‐chain fatty acids maintains intestinal eubiosis and reduces ethanol‐induced liver injury in mice. Gastroenterology 2015; 148: 203–14 e16. 25239591 10.1053/j.gastro.2014.09.014 PMC4274236

[34] Lamprecht M , Bogner S , Schippinger G , *et al* Probiotic supplementation affects markers of intestinal barrier, oxidation, and inflammation in trained men; a randomized, double‐blinded, placebo‐controlled trial. J Int Soc Sports Nutr 2012; 9: 45. 22992437 10.1186/1550-2783-9-45 PMC3465223

[35] Pascual S , Such J , Esteban A , *et al* Intestinal permeability is increased in patients with advanced cirrhosis. Hepatogastroenterology 2003; 50: 1482–6. 14571769

[36] Tripathi A , Lammers KM , Goldblum S , *et al* Identification of human zonulin, a physiological modulator of tight junctions, as prehaptoglobin‐2. Proc Natl Acad Sci USA 2009; 106: 16799–804. 19805376 10.1073/pnas.0906773106 PMC2744629

[37] Fukui H . Gut‐liver axis in liver cirrhosis: how to manage leaky gut and endotoxemia. World J Hepatol 2015; 7: 425–42. 25848468 10.4254/wjh.v7.i3.425 PMC4381167

[38] Loguercio C , Federico A , Tuccillo C , *et al* Beneficial effects of a probiotic VSL#3 on parameters of liver dysfunction in chronic liver diseases. J Clin Gastroenterol 2005; 39: 540–3. 15942443 10.1097/01.mcg.0000165671.25272.0f

[39] Besselink MG , van Santvoort HC , Buskens E , *et al* Probiotic prophylaxis in predicted severe acute pancreatitis: a randomised, double‐blind, placebo‐controlled trial. Lancet 2008; 371: 651–9. 18279948 10.1016/S0140-6736(08)60207-X

[40] Stadlbauer V . Immunosuppression and probiotics: are they effective and safe? Beneficial Microbes 2015; 6: 823–8. 26287986 10.3920/BM2015.0065

[41] Bischoff SC , Barbara G , Buurman W , *et al* Intestinal permeability – a new target for disease prevention and therapy. BMC Gastroenterol 2014; 14: 189. 25407511 10.1186/s12876-014-0189-7 PMC4253991

[42] Stadlbauer V , Davies NA , Wright G , Jalan R . Endotoxin measures in patients' sample: how valid are the results? J Hepatol 2007; 47: 726–7; author reply 7‐3. 17850915 10.1016/j.jhep.2007.08.001

[43] Horvath A , Leber B , Schmerboeck B , Stadlbauer V . Endotoxinmessung bei Leberzirrhose: Ein Methodenvergleich. Z Gastroenterol 2015; 53: P92.

