# Supplementary material for: Randomised clinical trial: the effects of a multispecies probiotic vs. placebo on innate immune function, bacterial translocation and gut permeability in patients with cirrhosis
Source: Aliment Pharmacol Ther. 2016 Sep 4;44(9):926–35. doi: 10.1111/apt.13788 (PMC5053220; doi:10.1111/apt.13788)
Supplement: Supplementary file 1 — Data S1. Rationale for per protocol analysis and additional methods. Table S1. Patients' characteristics and routine laboratory measurements for liver cirrhosis before, during and after intervention with a multispecies probiotic for the probiotic and placebo group compared to healthy controls. Table S2. Anti‐microbial molecules, acute‐phase proteins and serum killing incapacity before, during and after probiotic intervention for the probiotic and placebo group compared to healthy controls. Table S3. Parameters of endotoxin binding and gut permeability before, during and after probiotic intervention for the probiotic and placebo group compared to healthy controls. Figure S1. Effects of a multispecies probiotic on innate immune function before, during and after intervention for test groups compared to healthy controls. A+B. Oxidative burst profile of neutrophils during probiotic or placebo administration; C. Phagocytic capacity of monocytes; D. Inactive neutrophils; E. Serum killing incapacity of alcoholic cirrhotics; F. Change in serum killing incapacity of alcoholic cirrhotics after 6 months of intervention. *Significant differences to healthy controls; #significant differences to according baseline; significance level 0.05. Figure S2. Gut permeability and bacterial translocation before, during and after intervention for test groups compared to healthy controls. A, B. Mannitol and lactulose recovery in urine after triple sugar ingestion. C. Lactulose–mannitol ratio. D. Calprotectin in stool. E. Diamine oxidase in serum. F. Endotoxin in serum. *Significant differences to healthy controls; #significant differences to according baseline; ^significant changes between indicated groups; significance level 0.05. Figure S3. A. Formation of low and high phagocytic neutrophil population as used to calculate phagocytic capacity. B. Changes in Child–Pugh score from baseline to end of treatment in probiotic and placebo group; thickness of bars corresponds to number of patients w [file APT-44-926-s001.docx]

Supplementary information

Rationale for per protocol analysis

Since intention to treat analysis (ITT) gave the same significant changes (neopterin and neutrophil oxidative burst) within the groups as per protocol analysis (PPA) we decided to show PPA in the presented study. Although the baseline values of liver function, albumin and neutrophil count would be better balanced with ITT, we find that the imputation of values for the ITT, which is necessary almost exclusively in the placebo group (11 versus 1 dropout), would mask the natural progression of the disease. We substituted missing values with last observation carried forward, a rather conservative method in a chronic progressive disease. Dropout patients in this study refused to take the probiotic/placebo and did not consent to any further examinations. Therefore, we do not have access to clinical data or samples after the patients stopped the medication. Most of the dropout patients stopped the intervention between baseline and the study visit after 3 months (n=5) or between the visits after 3 and 6 months (n=4). One parameter would yield 48 data points for 12 dropouts. Only 45% of 48 points were actually measured and therefore, more than half would have to be imputated.

Sample preparation

Peripheral venous blood was aseptically collected at baseline and after three, six and twelve months into pyrogen-free tubes (VACUETTE®, Greiner Bio-One, Kremsmuenster, Austria) and kept at 4°C. For harvesting plasma blood was centrifuged at 2000xg for 10 min, for serum it was kept at room temperature for 30 minutes, and then centrifuged. After centrifugation, plasma or serum was aliquoted under pyrogen-free conditions into non-pyrogenic cryotubes (Eppendorf, Hamburg, Germany) and stored at -80°C until further analysis. Differential sugar absorption tests and stool sampling were performed every six months. Thiomersal was added to urine samples (1mg/ml) prior to storage at -80°C. Blood and stool samples of patients and controls were analysed in batches randomized into groups to correct for lot differences.

Participants were asked to complete a self-administered food frequency questionnaire about the usual dietary intake of 17 different groups of food and beverages to document possible changes in dietary habits.

*Phagocytosis assays*

Phagocytosis was assessed by Phagotest® (Glycotope, Heidelberg, Germany), a flow cytometric analysis using FITC-labelled *E. coli* bacteria. A novel strategy of analysis was used to determine phagocytic capacity, taking into account distinct populations of neutrophils. As phagocyte dysfunction progresses the proportion of inactive cells grows and a separate fraction with less phagocytic activity (low phagocytic) begins to form (Figure S1). Phagocytic capacity sums up the weighted geometric mean of fluorescence intensity (GMFI) of both active populations (low and high phagocytic) and is calculated as follows:

$$PC=hp \left( GMFI \right)\times hp \left( rs \right)+lp \left( GMFI \right)\times lp (rs)$$

(hp…high phagocytic, lp…low phagocytic, rs…relative share)

In addition, percentages of inactive neutrophils were analysed.

*Oxidative burst function*

The Phagoburst® kit (Glycotope, Heidelberg, Germany) was used as per manufacturer’s instruction to determine activation and burst profiles of neutrophils by flow cytometric analysis.

*Endotoxin measurement*

HEK-Blue™ LPS Detection Kit (Invivogen, San Diego, USA) with adapted protocol was used. In brief, cells were cultured in 24-well plates (5x10^4^cells/well). After 24 hours medium was discarded and replaced with samples/endotoxin standards and detection medium. Cells were incubated for 21 hours at 37°C and colour intensity was measured at a wave length of 650nm.

*Gut permeability markers*

Ready to use solid-phase sandwich ELISA were used to determine serum levels of diamine oxidase (Immundiagnostik AG, Bensheim, Germany), plasma levels of soluble cluster of differentiation 14 (sCD14) (R&D Systems, Minnesota, USA) and lipopolysaccharide binding protein (LBP) (Hycult, Uden, Netherlands) as well as zonulin and calprotectin in stool (Immundiagnostik AG, Bensheim, Germany). All tests were performed according to manufacturers’ instructions.

Differential sugar absorption

After overnight fasting first urine was collected and analysed as well as the collected urine over 5 hours after oral sugar challenge with 20g sucrose, 10g lactulose and 5g mannitol. Methanol, sodium phosphate, dibasic (Na_2_HPO_4_), sodium hydroxide, hydrochloric acid (32 % m/v), and sodium azide (NaN_3_) were obtained from VWR International (Darmstadt, Germany), 3(trimethylsilyl)propionic acid-2,2,3,3-d4 sodium salt (TSP) from Alfa Aesar (Karlsruhe, Germany), deuterium oxide (D_2_O) from Cambridge Isotope laboratories, Inc. (Tewksbury, MA). Phosphate buffer solution was lyophilized and redissolved in D_2_O. For NMR measurements 200µL urine was mixed with 300µL phosphate buffer solution. All NMR experiments were performed at 310K on a Bruker Avance III 500 MHz spectrometer equipped with a TXI probe head. The 1D CPMG (Carr−Purcell−Meiboom−Gill) pulse sequence (cpmgpr1d, 73728 points in F1, 12019.230 Hz spectral width, 128 transients, recycle delay 4 s), with water suppression using presaturation, was used for 1H 1D NMR experiments. Metabolite reference chemical shifts were taken from the Madison-Qingdao Metabolomics Consortium Database (<http://mmcd.nmrfam.wisc.edu/>) and all metabolites were cross-checked using reference compounds. Bruker Topspin version 3.1 and MestReNova version 10.0 software packages were used for NMR data acquisition, processing, and analyses. Metabolite concentrations were determined using TSP as internal standard.

*Cytokines*

ProcartaPlex™ multi-plex-assay (eBioscience, Vienna, Austria) for interleukin-1beta (IL-1β), interleukin-6 (IL-6), interleukin-8 (IL-8), interleukin-10 (IL-10) and tumour necrosis factor (TNF)-alpha was performed in sodium-citrate plasma according to manufacturer’s instructions.

*Serum antimicrobial activity*

A ready to use solid-phase sandwich ELISA was used to determine serum levels of neopterin (BRAHMS Diagnostics, Hennigsdorf, Germany). Serum bacterial killing incapacity was assessed by challenging 100 µl of serum with approximately 10^7^ CFU *E.coli (XL1blue)*. After 20min incubation at 37°C, 100µl suspension were plated on Luria-Bertani-agar (Sigma-Aldrich, St. Louis, USA) and cultured overnight at 37°C. Colony forming units were counted and compared. Bacteria were grown by inoculating 500µl overnight culture into 100ml fresh LB-broth (Sigma-Aldrich, St. Louis, USA) and incubated for 2 hours at 37°C. Optical density was used to estimate bacterial count and plating of serial dilutions for confirmation.

Table S1 **Patients characteristics and routine laboratory measurements for liver cirrhosis before, during and after intervention with a multispecies probiotic for the probiotic and placebo group compared to healthy controls.**

|  |  | Patients:  Probiotics (n=44) and Placebo (n=36) | | | | Controls (n=51) |
| --- | --- | --- | --- | --- | --- | --- |
|  | group | baseline | 3 months of intervention | end of intervention (6 months) | end of observation (12 months) |  |
| **HE (no/mild)** | **probiotics** | 39/5 | 40/4 | 38/6 | 40/4 | - |
|  | **placebo** | 35/1 | 34/2 | 34/2 | 34/2 |  |
| **Ascites (no/mild)** | **probiotics** | 40/4 | 41/3 | 37/7 | 38/6 | - |
|  | **placebo** | 32/4 | 34/2 | 33/3 | 34/2 |  |
| **GGT (U/l)** | **probiotics** | 125.0 (64.5; 234.8)^#^ | 107.0 (49.8; 246.0) | 114.5 (52.8; 205.8) | 110.5 (58.0; 198.3) | 20 (14.5; 31) |
|  | **placebo** | 107.5 (49.75; 175.3)^#^ | 111.0 (45.5; 179.3) | 122.0 (47.3; 192.0) | 109.5 (51.8; 244.0) |  |
| **TG (mg/dl)** | **probiotics** | 73.0 (55.0; 96.3) | 74.0 (62.8; 109.0) | 74.5 (62.5; 104.5) | 73.0 (57.0; 110.0) | 82.0 (68.0; 112.5) |
|  | **placebo** | 91.0 (69.8; 112.0) | 80.0 (65.0; 113.3) | 74.5 (63.0; 105.8) | 95.0 (59.8; 118.0) |  |
| **Neutrophils (10^6^/l)** | **probiotics** | 2.5 (2.0; 3.5)^#^ | 2.7 (2.0; 3.4) | 2.7 (1.8; 4.0) | 2.7 (1.9; 3.6) | 3.7 (3.0; 4.8) |
|  | **placebo** | 2.9 (2.5; 3.6) | 3.1 (2.4; 4.1) | 2.6 (2.2; 4.5) | 2.9 (2.2; 4.0) |  |
| **Monocytes (10^6^/l)** | **probiotics** | 0.4 (0.3; 0.5) | 0.4 (0.3; 0.6) | 0.4 (0.3; 0.6) | 0.4 (0.3; 0.6) | 0.5 (0.4; 0.6) |
|  | **placebo** | 0.5 (0.3; 0.6) | 0.5 (0.3; 0.5) | 0.5 (0.4; 0.6) | 0.5 (0.3; 0.5) |  |

HE, hepatic encephalopathy; GGT, gamma glutamyl transferase; TG, triglycerides; data is given in median (Q1, Q3) # significant difference compared to control group; significance level 0.05

Table S2 **Antimicrobial molecules, acute phase proteins and serum killing incapacity before, during and after probiotic intervention for the probiotic and placebo group compared to healthy controls.**

|  |  | Patients:  Probiotics (n=44) and Placebo (n=36) | | | | Controls (n=51) |
| --- | --- | --- | --- | --- | --- | --- |
|  | group | baseline | 3 months of intervention | end of intervention (6 months) | end of observation (12 months) |  |
| **CRP (mg/l)** | **probiotics** | 2.0 (0.9; 4.7) | 1.9 (1.0; 5.2) | 2.5 (0.8; 5.2) | 3.2 (0.9; 4.8) | 1.4 (0.8; 2.0) |
|  | **placebo** | 2.6 (1.2; 3.4)^$^ | 1.8 (0.7; 3.4) | 2.2 (0.9; 4.1) | 2.6 (1.1; 4.0) |  |
| **Ferritin (mg/dl)** | **probiotics** | 160.0 (59.3; 357.8) | 153.0 (50.3; 247.8) | 152.5 (52.5; 104.5) | 154.0  (54.3; 246.0) | 117.5  (60.3; 218.5) |
|  | **placebo** | 97.5 (55.3; 221.3) | 92.0 (43.8; 166.0) | 90.5 (47.3; 154.5) | 96.0 (40.5; 179.0) |  |
| **Fibrinogen (mg/dl)** | **probiotics** | 266.5 (216.3; 313.5)^#^ | 278.0 (212.5; 325.3) | 261.0 (214.3; 316.3) | 262.5 (203.5; 321.0) | 302.0 (254.0; 328.0) |
|  | **placebo** | 277.0 (241.3; 308.5) | 283.5 (249.5; 325.0) | 292.0 (260.3; 318.5) | 283.0 (240.3; 329.3) |  |
| **Nitrite (µmol/l)** | **probiotics** | 9.5 (5.2; 15.2) ^#$^ | 8.0 (6.2; 13.6) | 9.1 (6.0; 14.7) | 10.9 (6.7; 21.3) | 19.4 (9.5; 32.4) |
|  | **placebo** | 10.6 (6.0; 18.4) ^#$^ | 9.1 (7.9; 14.3) | 7.9 (5.7; 13.7) | 14.0 (9.3; 19.8) |  |
| **Serum calprotectin (ng/ml)** | **probiotics** | 432.2 (326.6; 896.6) | 445.7 (240.1; 779.4) | 460.9 (326.5; 824.7) | 356.6 (214.0; 808.2) | 536.6 (334.0; 779.0) |
|  | **placebo** | 627.9 (325.2; 798.5) | 456.2 (301.1; 847.5) | 476.8 (297.3; 953.4) | 482.6 (323.1; 626.2) |  |
| **Serum zonulin (ng/ml)** | **probiotics** | 39.8 (23.1; 53.7)^#^ | 39.8 (23.3; 55.1) | 34.1 (22.0; 51.9) | 37.8 (22.9; 58.2) | 56.0 (48.0; 66.0) |
|  | **placebo** | 46.2 (26.6; 63.5) | 44.8 (27.3; 63.0) | 44.7 (31.9; 61.0) | 44.3 (28.5; 63.6) |  |
| **Killing incapacity (surviving CFU)** | **probiotics** | 359 (86; 1238)^#^ | 485 (139; 1914) | 366 (87; 877) | 370 (94; 2161) | 55 (31;146) |
|  | **placebo** | 184 (42; 1289)^#^ | 155 (67; 1538) | 341 (79; 774) | 234 (53; 887) |  |

CFU, colony forming units; #significant difference compared to control group; $significant change over time; significance level 0.05

Table S3 **Parameters of endotoxin binding and gut permeability before, during and after probiotic intervention for the probiotic and placebo group compared to healthy controls.**

|  |  | Patients: Probiotics (n=44) and Placebo (n=36) | | | | Controls (n=51) |
| --- | --- | --- | --- | --- | --- | --- |
|  | group | baseline | 3 months of intervention | end of intervention (6 months) | end of observation (12 months) |  |
| **sCD14 (µg/ml)** | **probiotics** | 1.9 (1.6; 2.2) | 2.0 (1.6; 2.3) | 1.9 (1.6; 2.3) | 1.9 (1.6; 2.2) | 1.7 (1.6; 2.0) |
|  | **placebo** | 2.0 (1.7; 2.3) | 1.9 (1.8; 2.4) | 2.0 (1.7; 2.2) | 2.0 (1.7; 2.4) |  |
| **LBP (µg/ml)** | **probiotics** | 18.5 (13.4; 25.8) | 16.2 (12.0; 19.0) | 17.4 (14.5; 23.6) | 18.8 (15.7; 22.3) | 16.5 (13.4; 21.1) |
|  | **placebo** | 18.6 (13.3; 34.5) | 16.9 (14.5; 20.7) | 17.9 (13.6; 22.1) | 17.0 (14.1; 21.1) |  |
| **Zonulin (ng/ml)** | **probiotics** | 82.7 (66.9; 105.5) | - | 80.6 (68.1; 96.5) | 75.7 (62.6; 100.3) | 73.9 (27.6; 86.7) |
|  | **placebo** | 71.4 (55.3; 93.4) | - | 73.7 (59.1; 88.9) | 71.3 (58.4; 88.1) |  |
| **Sucrose recovery (%)** | **probiotics** | 0.007 (0.0; 0.10) | - | 0.002 (0.0; 0.09) | 0.0 (0.0; 0.09) | 0.045 (0.0; 0.10) |
|  | **placebo** | 0.057 (0.0; 020) | - | 0.008 (0.0; 0.10) | 0.040 (0.0; 0.17) |  |

sCD14, soluble cluster of differentiation; LBP, LPS binding protein; data is given in median (Q1, Q3); significance level 0.05





Figure S1: Effects of a multispecies probiotic on innate immune function before, during and after intervention for test groups compared to healthy controls. A+B. Oxidative burst profile of neutrophils during probiotic or placebo administration; C. Phagocytic capacity of monocytes; D. Inactive neutrophils; E. Serum killing incapacity of alcoholic cirrhotics; F. Change in serum killing incapacity of alcoholic cirrhotics after six months of intervention; *significant differences to healthy controls; #significant differences to according baseline; significance level 0.05





Figure S2: Gut permeability and bacterial translocation before, during and after intervention for test groups compared to healthy controls. A-B. Mannitol and lactulose recovery in urine after triple sugar ingestion; C. Lactulose-mannitol ratio; D. Calprotectin in stool; E. Diamine oxidase in serum; F. Endotoxin in serum; *significant differences to healthy controls; # significant differences to according baseline; ^ significant changes between indicated groups; significance level 0.05


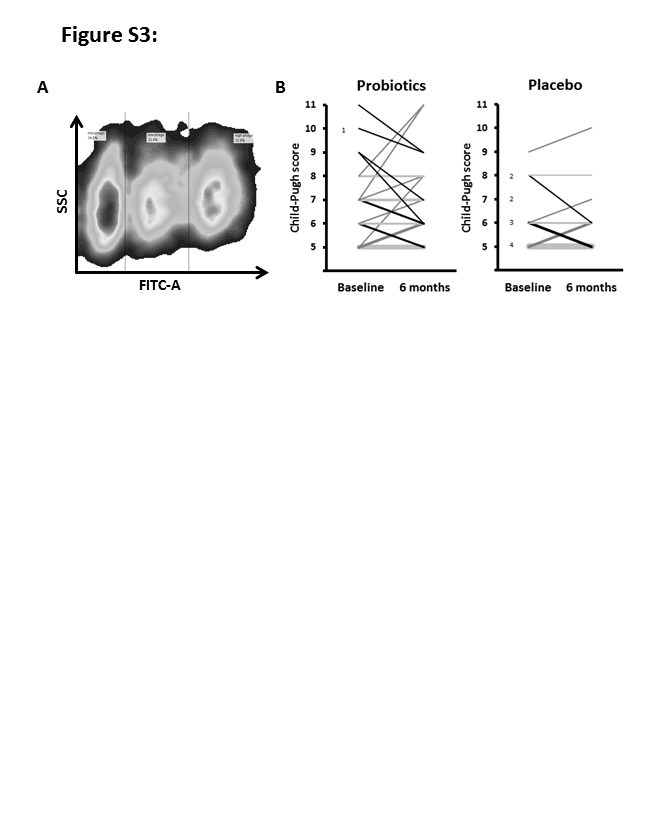


Figure S3: A. Formation of low and high phagocytic neutrophil population as used to calculate phagocytic capacity; B. Changes in Child-Pugh score from baseline to end of treatment in probiotic and placebo group; thickness of bars corresponds to number of patients with respective changes.
